# Factors associated with the severity and complication of patients with malaria hospitalized between 2009 and 2013 in three municipalities of Colombia, case control study

**DOI:** 10.1186/s12936-016-1554-5

**Published:** 2016-10-19

**Authors:** Jesica F. Ramírez, Beatriz Porras, Elizabeth Borrero, Sandra P. Martínez

**Affiliations:** Fundación Santa Fe de Bogotá Centro de Estudios e Investigación en Salud-CEIS, Carrera 7 B # 123-90, Piso 3, Bogotá, Colombia

**Keywords:** Colombia, Hepatic dysfunction, Jaundice, Malaria, Severity, Thrombocytopaenia

## Abstract

**Background:**

Malaria worldwide annual reported cases range between 250 and 500 million and nearly half a million deaths are reported every year. Colombia has a vast expanse of territory with environmental and social conditions conducive to malaria transmission, which is the reason why it has second place in Latin America for the number of cases of malaria.

**Methods:**

This is a retrospective, paired, case–control study that compares patients with severe malaria and malaria patients without mention of complication. Medical records of patients with confirmed malaria diagnosis were reviewed. The objective of this study was to identify factors associated with complicated malaria in three municipalities of Colombia during the period 2009–2013.

**Results:**

A total sample of 180 medical records was collected, 60 corresponding to cases and 120 to controls following a 1:2 ratio. From the total sample, 88.33 % (159) of subjects were originally from Tumaco, 7.78 %, most of the patients of the study (104) were diagnosed and treated in 2013. Among the laboratory findings, the platelet count was found to have statistically significant differences between cases and controls; this was also true for total bilirubin value and transaminases. The most prevalent severity finding was clinical jaundice, followed by elevated bilirubin (25 %) and elevated transaminases (44.4 %). Thrombocytopenia was found in 11/44 cases (20 %) and only five of 60 patients (8.3 %) presented severe anaemia. The multivariate analysis showed that a platelet count <100,000 and clinical jaundice not associated with organ failure, were the two variables associated with severe malaria in the patients described.

**Conclusions:**

Most of the cases studied, despite meeting criteria of severity, were shown not to be as morbid or severe as in other countries. Clinical jaundice and thrombocytopaenia are associated with severe malaria, and they can be used by general practitioners or primary care physicians to promptly identify patients who may have severe malaria.

## Background

Although other Plasmodium species can cause severe disease and death, most of these outcomes are reported in *Plasmodium falciparum* infections. Annual statistics of malaria cases worldwide range between 250 and 500 million, and nearly half a million deaths are reported every year. Severe cases and deaths from malaria occur mainly in the poorest regions of countries affected with social exclusion [1].

Colombia has a vast expanse of territory with environmental and social conditions conducive to malaria transmission, which is the reason why it has earned second place in the number of cases of malaria in Latin America. In 2010 an estimate of more than one million cases of malaria and 1100 deaths from malaria was made in the South and Central American region. Brazil reported the highest number of cases (54.4 %), followed by Colombia (13.1 %) and Venezuela (9.3 %). The largest number of deaths from malaria reportedly occurred in Brazil, Colombia and Dominican Republic [2].

The major complications of malaria described in literature are cerebral malaria, pulmonary oedema, acute renal failure, severe anaemia, bleeding, acidosis and hypoglycaemia [3]. Cerebral malaria is the presentation of the disease which most frequently leads to mortality [4]. It is of utmost importance to identify the red flags and severity-associated symptoms in patients with malaria, so these patients can be promptly diagnosed and provided with the medical care necessary to prevent fatality. This study aims to identify factors associated with severity and complications of malaria in three municipalities of the Colombian Pacific over a 5-year period.

## Methods

This is a retrospective, matched, case–control study that compares patients with severe malaria and malaria patients without mention of complication. The data used in this study were collected from medical records of patients with confirmed malaria diagnosis, hospitalized between 2009 and 2013, in four different hospitals located in endemic areas of the Colombian states of Nariño and Valle del Cauca, covering the municipalities of Tumaco, Buenaventura and Cali. The total data collection time was 14 months, starting in 2012.

### Sample size definition

Considering the findings of the study performed by Tobon et al. [5], the proportion of patients with respiratory distress was used to calculate the sample size. The proportion of patients with respiratory distress considered for cases was 38 and 14 % for controls. A case/control ratio of 1:2, confidence level of 95 % and power of 90 % were used to design the study. The sample size required for the study following these parameters was 60 cases and 120 controls.

### Matching process

Matching of cases and controls was performed considering year of malaria diagnosis, municipality of origin and *Plasmodium* species. Matching by year of presentation and municipality was done to assure that the patient base population of cases and controls remained constant. The area where the study was conducted has a population that is constantly changing and moving in between regions due to their economical activities, such as mining, and the effect of the armed conflict that affects the area. The biggest proportion of cases of severe malaria is reported in the young adult population. Given the fact that malaria presentations change importantly with age, a decision was made to not include the age in the matching process so further analysis could be made from the outcomes of the study related with the age. A bivariate analysis was conducted to determine if the variable age was confounding factor or a factor associated with severity. Due to the lack of information regarding previous malaria episodes in the medical records reviewed, matching by this variable was not possible.

### Data collection

A detailed review of the National System for Public Health Surveillance (SIVIGILA) databases from 2009 to 2012 was performed to identify the medical records of cases and controls available to review in each municipality. Once the data were obtained, a formal request was sent to each institution to obtain access to the medical records. In order to obtain enough medical records of malaria patients, information from malaria public health notification forms located in the Public Health and Statistic Departments of the hospitals were also used.

An on-line instrument in the form of a questionnaire was designed as well as a guideline with definitions, inclusion and exclusion criteria, to be used by trained doctors to collect the data from the medical records in the designated areas. The instrument included: personal identification, past medical history, history of present illness, clinical characteristics, physical examination, diagnosis, paraclinical and parasitological results on admission, treatment, and case outcome. The instrument was accessible via email, using the SurveyMonkey^®^ tool and was filled using mobile devices with internet access to assure real-time accessibility and quality control of the data being collected.

To ensure the instrument was filled out correctly, three independent reviewers evaluated the quality of the information filled out for each medical record, using a constructed validation mesh interdisciplinary with the statistical unit, and provided feedback to the doctors when necessary to correct discrepancies.

### Inclusion criteria

Patients with signs or symptoms of malaria, with confirmed diagnosis of malaria infection with either *Plasmodium vivax* or *P. falciparum*, made by thick or thin smears and treated in the municipalities were included in the study.

### Exclusion criteria

Cases of sickle cell disease, viral or bacterial infections and/or other diseases that could explain the clinical presentation, as well as cases that originated from a municipality not included in the area of analysis, or cases that were transferred from another municipality to receive medical care were excluded.

### Case definition

Subjects hospitalized between 2009 and 2013, with diagnostic confirmation of *P. vivax* or *P. falciparum* via thick blood smear slides, and with one or more of the clinical or paraclinical criteria mentioned in the Colombian National Guidelines for severe malaria, were defined as cases, as shown in Table 1.

**Table 1:** Severity criteria for severe malaria

Clinical criteria	Laboratory criteria
Loss of consciousness or profound coma	Haemoglobinuria
Prostration; extreme weakness with inability to walk or sit without assistance	Hypoglycaemia (<60 mg/dL)
Inability to feed oneself	Metabolic acidosis (plasmatic bicarbonate <15 mmol/L)
Multiple seizures; more than 1 episode in 24 h	Hyperlactaemia (lactate acid <5 mmol/L)
Respiratory distress syndrome	Severe anaemia (haemoglobin <7 g/dL, haematocrit <21 %)
Circulatory collapse/shock; systolic arterial pressure <80 mmHg in adults and <50 mmHg in children	Hyperparasitaemia (>50,000 asexual parasites/μL) with the diagnosis of *P. falciparum*, or mixed infection with *P. vivax*
Clinical jaundice with signs of vital organ failure	Thrombocytopaenia (<50,000 mm^3^)
Spontaneous haemorrhage	Elevated transaminase (>80 IU)*
Pulmonary oedema evidenced through radiography	Elevated total bilirubin (>1.5 mg/dL) Renal insufficiency (serum creatinine level >1.5 mg/dL)

* Elevated transaminases >80 UI was the criteria used in this study; which differs from the OMS guideline of transaminases three times the normal range and the Colombian Guideline of transaminases >40 UI

The Colombian National Guidelines differ from the World Health Organization (WHO) guidelines, for severe malaria they define hepatic dysfunction as transaminase values >40 IU. According to medical literature and WHO, malarial hepatic dysfunction is defined as an elevation of three times the upper limit of normal transaminase values. An intermediate value of >80 IU was utilized in this study in order to analyse the possibility of proposing a new cut-off point for the Colombian guidelines, given the low prevalence of hepatic failure and hepatic dysfunction in Colombia [6].

### Control definition

Patients with clinical malaria diagnosis confirmed by thick or thin blood smear slides, proven to be infected with *P. falciparum* or *P. vivax,* that did not have any of the severity criteria mentioned for case definition and were proven to be infected and treated in the municipalities, were included in the study.

### Statistical analysis

In order to identify inconsistencies and extreme values in the dataset, an exploration of the variables included in the study was performed. A univariate descriptive analysis, including the demographic, clinical and laboratory data was performed using frequency tables and central tendency measures according to the type of variable.

Given the design of the study, paired case control study 1:2 ratio, paired by year of diagnosis, municipality and *Plasmodium* species, a Student’s t test was performed to analyse differences between case and control in the numeric variables, and McNemar’s test for categorical variables. Conditional logistic regression was used to calculate the odds ratio and 95 % confidence intervals to find association between independent variables and severe malaria. In order to find association between clinical factors and severe malaria, a multiple conditional logistic regression was performed using the variables that were found to be statistically significant (P < 0.05) or relevant in the bivariate analysis, controlling confounding factors and analysing possible interactions between variables. Verification that the statistical models complied with all the assumptions of the logistic regression was also performed.

Variables considered in the statistic bivariate analysis and statistical model were: gender, age, place of origin, duration of symptoms before admission, symptomatology on admission, presence of co-morbidities, days hospitalized, vital signs on admission, thick and thin blood smear slide results, physical examination on admission, medical treatment received, initial laboratory values, pregnancy status, previous history of malaria, and prior medical attendance before admission. Statistical analysis was processed using STATA^®^ version 12.

### Ethical considerations

Ethical approval was granted by the ethics committee that permitted access to patient medical history and data collection for analysis and conversion to a scientific manuscript.

## Results

The total sample collected for cases and controls was 180, 60 medical records corresponding to cases and 120 to controls, following a 1:2 ratio. From the total sample 88.33 % (159) of the subjects were originally from Tumaco from which 53 were cases and 106 were controls. 5.00 % (nine) from Cali, three were cases and six controls, and 6.66 % (12) from Buenaventura four were cases and eight controls. Regarding the year of medical consultation, six of the subjects were seen in the emergency room in 2009, 17 in 2010, 12 in 2011, 41 in 2012, and 104 in 2013.

Half of the malaria cases with confirmed severity criteria were women, and the other 50 % were men. For the control group, 46.67 % (56) were women and 53.33 % were men. As seen in Table 2, the adult population predominated both in the control and case groups.

**Table 2:** Demographic characteristics of the population

	Control		Case	
	N	%	N	%
*Age*				
<5	12	10	1	1.67
5–9	13	10.83	4	6.67
10–14	19	15.83	8	13.33
15–19	13	10.83	11	18.33
20–29	17	14.17	18	30
30–39	26	21.67	6	10
40–49	7	5.83	8	13.33
>50	13	10.83	4	6.67
*Sex*				
Male	64	53.33	30	50
Female	56	46.67	30	50
*Healthcare affiliation*				
Affiliated	112	93.34	54	90
Not affiliated	8	6.67	6	10

Regarding the general system of social security affiliation status, more than 90 % of the cases and controls were affiliated at the time of their medical attention, meaning that medical attention should be provided to them by law in any public health institution.

### Medical care

The average number of days hospitalized for the cases was of 4.1 days, (SD ± 1.77), the average days since onset of symptoms until first medical attention according to the information contained in the medical records was 5.72 days for cases (SD ± 4.45 days) and 5.60 for controls (SD ± 4.97 days). From the total sample, 71 % (43) of the cases and 45 % (54) of the controls were referred from other institutions where they received their first medical attention. Hospitals providing basic medical care, first-level institutions were found to be the main medical institutions 90.7 %.

Forty-four cases were confirmed to be *P. falciparum* (73 %) and 16 *P. vivax* (26 %). For controls, 87 were confirmed *P. falciparum* (72 %) and 33 *P. vivax* (27 %).

In the medical records, evidence was found that 27 % of the cases and 19 % of the controls took medication without prescription before admission to hospital. The most common medications found to be used by the patients were home-made remedies (12 %) and analgesics (87 %). From the total sample, 29 % of cases and 16 % of controls had a known medical condition at admission (co-morbidities), the most common being gastritis (10), high blood pressure (5), asthma (4), urinary tract infection (4), and diabetes (3).

### Physical examination at the time of admission

As shown in Table 3, there were no statistically significant differences between cases and controls regarding temperature, respiratory rate, heart rate, or blood pressure. In the neurologic examination, 98.33 % (59) of the cases were found to be alert at the time of admission compared to 100 % for the controls; 3.4 % (two) and 2.7 % (three) of the cases and controls, respectively, were found to have intolerance to the oral route; 37 % (20) of the cases and 9 % (nine) of the controls presented jaundice at the time of admission; 19 % (nine) of the cases presented physical signs of hepatomegaly compared to 4 % (three) for the controls; four (8 %) cases presented with splenomegaly at the time of admission. Dark urine was presented by 7/60 of cases and four of 120 controls, haemorrhagic manifestations were seen in two cases (3 %). The main haemorrhagic manifestation was epistaxis.

**Table 3:** Difference in the average of vital signs and its statistical significance thereof at the time of admission in cases and controls

Signs	Parameter	Case	Control	P
Temperature (°C)	Average	37.31	37.64	<0.11
	SD	1.36	1.23	
	Min	34.50	36.00	
	Max	40.20	40.50	
Heart rate	Average	91.90	92.79	<0.75
	SD	16.83	17.83	
	Min	63.00	60.00	
	Max	140.00	155.00	
Respiratory rate	Average	22.95	22.13	<0.36
	Variance	20.08	39.31	
	SD	4.48	6.27	
	Min	16.00	14.00	
	Max	40.00	63.00	
Systolic pressure	Average	104.17	109.72	<0.05
	Variance	186.52	296.23	
	SD	13.66	17.21	
	Min	60.00	63.00	
	Max	140.00	190.00	
Diastolic pressure	Average	66.44	68.39	<0.36
	Variance	180.68	115.10	
	SD	13.44	10.73	
	Min	20.00	40.00	
	Max	90.00	100.00	

P value using mean difference between sample data pairs

### Laboratory findings

Table 4 shows the laboratory findings at the time of admission; no significant differences were found between cases and controls regarding haemoglobin levels, haematocrit, leucocyte count or parasitic count. The platelet count was found to have a significant difference between cases and controls. Statistically significant differences were also found between the cases and controls hepatic function tests, the average total bilirubin value and aspartate aminotransferase (AST) and alanine aminotransferase (ALT) values were found to be different between the two groups.

**Table 4:** Difference in the mean average of laboratory findings and its statistical significance at the time of admission in cases and controls

Signs	Parameter	Case	Control	P
Haemoglobin	Average	11.99	11.84	<0.66
	SD	2.51	1.94	
	Min	6.20	7.20	
	Max	15.90	17.00	
	N*	59	109	
Haematocrit	Average	35.78	34.86	<0.40
	SD	7.09	5.94	
	Min	18.10	23.60	
	Max	45.47	48.00	
	N*	53	89	
Platelets	Average	118,010	179,407.77	<0.00
	SD	121,344	106,059.5	
	Min	11,800	17,800.0	
	Max	850,000	837,000.0	
	N*	55	103	
Total bilirubin	Average	3.41	0.97	<0.00
	SD	1.90	0.33	
	Min	1.40	0.19	
	Max	7.30	1.40	
	N*	16	13	
ALT	Average	98.85	28.00	<0.00
	SD	91.99	12.04	
	Min	10	10.00	
	Max	381	60.00	
	N*	27	33	
AST	Average	103.33	35.26	<0.00
	SD	87.59	13.43	
	Min	18	14.00	
	Max	377	69.00	
	N*	27	31	
Creatinine	Average	0.90	0.85	0.37
	SD	0.26	0.23	
	Min	0.50	0.48	
	Max	1.50	1.36	
	N*	37	54	
Glycaemia	Average	110,12	107,65	<0.77
	SD	25,01	45,48	
	Min	63	67	
	Max	169	350	
	N*	33	44	
Trophozoite count	Average	8,076.75	5,924.17	<0.32
	SD	15,795.4	7,409.47	
	Min	80	240	
	Max	77,600	49,120	
	N*	37	76	

P value using mean difference between sample data pairs

N* number of medical records with data available

### Severity findings

The most prevalent severity finding in the cases group was clinical jaundice as described above, followed by elevated bilirubin 15/15 cases (25 %) and elevated transaminases 12/27 (44 %). Thrombocytopaenia was found in 11/44 cases (20 %) and only five of 60 patients (8 %) presented severe anaemia.

Table 5 presents the severity signs at time of admission in the cases. In the bivariate analysis the variables that showed significant association were chills, dark urine, platelet count, clinical jaundice, and referral from a lower level facility to a higher level. Other variables, such as co-morbidities tachycardia, signs of respiratory distress, haemorrhagic manifestations, anaemia, showed association but were not significant. The multivariate analysis showed that a platelet count <100,000 and clinical jaundice were not associated with organ failure, and were the two variables associated with severe malaria in the patients described (Table 6).

**Table 5:** Factors associated with severity in malaria found in bivariated and multivariated analysis results

Variable	OR	Confidence interval		Adjusted OR	Confidence interval	
Remitted	3.79	1.75	8.21			
Chills	3.44	1.14	10.42			
Dark urine	3.49	1.02	11.95			
Platelet count <100,000	11.83	3.53	39.59	9.41	2.52	35.08
Clinical jaundice	4	1.72	9.24	3.85	1.43	10.34
Co-morbidities	1.93	0.87	4.26			
Tachycardia	1.36	0.63	2.91			
Temperature >38.7 °C	0.78	0.37	1.66			
Dehydration	0.75	0.34	1.63			
Signs of respiratory distress	3.23	0.58	17.99			
Haemorrhagic manifestations	8.58	0.98	74.9			
Anaemia	1.47	0.72	2.99			
Creatinine	1.23	0.4	3.77			
AST	1.77	0.28	11.21			
ALT	5.46	0.59	4.94

**Table 6:** The signs of severity found in cases at time of admission

Severity signs at the time of admission	Present		Absent	
	N	%	N	%
Loss of consciousness or profound coma	2	3.30	58	96.70
Prostration; extreme weakness with inability to walk or sit without assistance	No data available	No data available	60	100
Inability to feed oneself	No data available	No data available	60	100
Multiple seizures; more than 1 episode in 24 h	1	1.70	59	98.30
Respiratory distress syndrome	3	5.00	57	95.00
Spontaneous haemorrhage	2	3.30	58	96.30
Circulatory collapse/Shock SAP <80 mmHg (adults) SAP <50 mmHg (children)	4	6.70	56	93.30
Pulmonary oedema evidenced through radiography	1	1.7	59	98.30
Jaundice	33	27.5	87	72.5
Haemoglobinuria	1	1.70	59	98.30
Severe anaemia (Haemoglobin <7 g/dL, haematocrit <21 %)	5	8.30	55	91.70
Hypoglycaemia (glycaemia <60 mg/dL)	2	3.30	58	96.70
Metabolic acidosis (plasmatic bicarbonate <15 mmol/L)	No data available	No data available	No data available	No data available
Hyperlactaemia (lactate acid >5 mmol/L)	No data available	No data available	No data available	No data available
Renal insufficiency (serum creatinine level >1.5 mg/dL)	6	10.00	54	90.00
Hyperparasitaemia (>50,000 asexual parasites/μL)	4	6.70	56	96.30
Thrombocytopaenia <50,000 mm^3^	11	20.0	44	80.0
Elevated transaminase AST >80 IU	12	44.4	15	55.5
Elevated trasaminase ALT >80 IU	12	44.4	15	55.5
Elevated total bilirubin (>1.5 mg/dL)	15	25.0	No data available	No data available

Regarding the neurological signs and symptoms, only one patient presented with loss of consciousness, and one patient presented with multiple seizures; no data was available regarding the presence of signs of prostration or inability to feed oneself. Few patients presented with respiratory complications: three patients showed signs of respiratory distress syndrome and one had evidence of pulmonary oedema through radiography.

There was no data available to determine the presence of metabolic acidosis, or hyperlactataemia. Further information about severity findings at the time of admission is presented in Table 6.

## Discussion

Malaria mortality and severity in Colombia, especially in the Pacific region, has maintained a decreasing trend given the improvement in knowledge of the population and from the health providers regarding early symptoms and malaria prevention, as well as more coverage and access to early diagnosis and treatment [7].

During the last decade the Pacific area has received help from the Global Fund for AIDS, the US National Institute of Allergy and Infectious Diseases, the Colombian Ministry of Social Protection and National Institute of Health, which has significantly contributed to a reduction in malaria transmission and mortality, given education and training of health personnel and communities [7, 9]. However, it has been described in the literature and confirmed in this study that patients are diagnosed with malaria approximately 72 h after symptom onset, which may be explained by the difficulties of access to healthcare facilities in the area, and to poverty and armed conflict present in areas of high transmission, which is adversely affecting malaria control efforts [7, 8].

In this study, the adult population was found to be affected in greater proportion than other age groups: only 11 % of the sampled patients were under 5 years of age, as has previously been described in the literature [8, 9]. The working adult, economically active population is the most affected, given that the area is known for its mining and fish farming activities with the presence of permanent mosquito breeding sites [7].

Even though the patients analysed in this study had severity criteria that classified them as having severe or complicated malaria, the severity of the signs and symptoms was not as high as described in areas of Africa or Asia. The literature proposes clinical immunity to malaria in low-transmission settings to be one of the reasons for this phenomenon [9]. The Colombian literature reports that 17 % of the patients have presented multiple malaria episodes [8, 10]. Although this study aimed to describe this situation, the medical records reviewed did not have sufficient data, 43/60 cases and 76/120 of the controls did not have information available, which is the reason why this variable was not included in the multivariate analysis.

In Colombia, the diagnosis of severe malaria before 2010 was made using the criteria established by the WHO; however in this year the Colombian Ministry of Health created a document based on the literature to adapt the criteria for the Colombian population, which included more conservative criteria, such as anaemia (haemoglobin <7 g/dL in adults and children), renal dysfunction (serum creatinine >1.5 mg/dL), severe thrombocytopaenia (≤50,000 platelets/μL), and hyperparasitaemia (>50,000 parasites/μL), and this is currently been used by healthcare professionals in the country to diagnose severe malaria [8]. However, most studies published on malaria in Colombia use a combination of both WHO and Colombian guidelines to analyse their patients [9]. Colombian guideline criteria were used in this study with an adaptation of the transaminase level two times the normal, aiming to analyse the extent of hepatic dysfunction in the population. However, data regarding liver function test were limited, and no statistically significant difference was found in patients with altered hepatic function and severity of malaria. Factors found to be associated with severe malaria in this study were platelet count <100,000 and clinical jaundice without evidence of organ failure. Low platelet count, hepatic failure and severe anaemia, among others, have been reported in Colombian studies to be the main malaria complications in Colombia [8].

As described in the literature, thrombocytopaenia is common in severe malaria cases in Colombia; in most cases a mild to moderate thrombocytopaenia (50,000–150,000 platelets/μL) is seen and generally does not represent a risk of spontaneous bleeding [9]. In the Colombian national malaria guidelines, severe thrombocytopaenia is considered when platelet counts are less than 50,000/mm^3^ [6], which was found in 11 cases of the present study. Platelet counts of less than 100,000/mm^3^ were found to be a factor associated with severe malaria, and were described in 30 cases, with an odds ratio (OR) of 9.41 IC 95 % (2.52; 35.08).

Jaundice was another factor found to be associated with severe malaria in the present study, and it is important to point out that cases found to have jaundice had no organ damage or dysfunction, which suggests that jaundice is a relevant clinical sign in patients where severe malaria is suspected [6, 11].

Severe anaemia significantly contributes to malaria mortality. The literature reports that mild to moderate anaemia in the majority of the population is related to malnutrition or intestinal parasites and other conditions related to poverty. In this study, 85 patients presented with haemoglobin levels <12 g/dL, considered as anaemia for both men and women, but two patients presented with haemoglobin levels less than 7 g/dL, i.e., severe anaemia and a criterion of severe malaria [12].

Cerebral malaria was not observed in this study, and its incidence has been reported to be low in Colombia [9].

Limited data are available regarding the association between malaria severity and co-morbidities, as opposed to infectious co-morbidities. In this study, no association was found between them: OR 1.93 CI (0.87; 4.26). In the population studied, ten cases and 17 controls were found to have self-medicated with home-made remedies and over-the-counter analgesics before seeking medical attention. This needs to be further analysed in other studies, given its possible relation to the time of consultation of patients.

Differences between species regarding the prevalence of thrombocytopaenia, cerebral malaria and other severity criteria have been described in the literature, however no conclusion or analysis between species was made in this study given that a match by species was made as part of the design of the study.

The literature regarding the characteristics of complicated malaria in Colombia is limited, there is a broad spectrum of clinical manifestations, and there is no information regarding multiple infections and the development of immunity, association of severity signs with different *Plasmodium* species, characterization of the affected population, or the treatment options available. Most studies available are retrospective and developed in the Pacific area. More studies that describe the current situation and contribute to characterization of complicated malaria in patients in Colombia are needed [8, 9].

It is of utmost importance for health personnel specially physicians to identify the red flags for severe malaria, described in the Colombian literature as danger signs. Changes in mental status, seizures, jaundice, bleeding among others have been described as danger signs and should be detected early in order to prevent complications and mortality associated with malaria. In this study, jaundice was found to be the main sign associated with severity in the population studied, and it is well-recognized as a danger sign in the Colombian literature.

Even though platelet count severity criteria for the Colombian Guidelines and WHO are 50.000 and 20.000/µL, respectively, in this study having platelet counts less than 100.000/µL was found to be a significant danger sign [13].

## Limitations

The research was rigorous, following the methodology described and as mentioned there were three reviewers of the data, however one of the weaknesses is that it was a retrospective study, there were difficulties in collecting data, there was a lack of quality of medical records by omission of relevant clinical data in patients attending consultation for malaria. The data collection was first programmed to be done in specific municipalities selected in Colombia, based in the information of areas of higher transmission but due to administrative issues this was not possible. The data collection was limited to the municipality of Tumaco where medical records were available. Buenaventura Hospital was closed down during the research process which made it impossible to access medical records. In Cali, most of the patients were found to have non-complicated malaria, and there were few medical records that had severity criteria and were considered cases to pair with the controls.

## Conclusion

The data collected form the medical records reviewed showed that most cases of malaria that occur in the population, despite meeting criteria of severity, have been shown to be not as morbid or severe as in other countries. The study showed that jaundice and thrombocytopaenia are associated with severe malaria, and they can be used with other signs and symptoms by general practitioners or primary care physicians to promptly identify patients to may progress towards clinical complications.

## Abbreviations

SD: standard deviation
AST: aspartate aminotransferase
ALT: alanine aminotransferase
WHO: World Health Organization
